# On the measurement of glomerular filtration rate: An odyssey into the *milieu intérieur*

**DOI:** 10.1113/EP092699

**Published:** 2025-03-25

**Authors:** Anders Møller Greve, Anna Agnes Lytzen, Hrefna Sæunn Einarsdóttir, Michael Perch, Ronan M. G. Berg

**Affiliations:** ^1^ Department of Clinical Biochemistry Copenhagen University Hospital – Rigshospitalet Copenhagen Denmark; ^2^ Centre for Physical Activity Research Copenhagen University Hospital – Rigshospitalet Copenhagen Denmark; ^3^ Department of Clinical Physiology and Nuclear Medicine Copenhagen University Hospital – Rigshospitalet Copenhagen Denmark; ^4^ Department of Clinical Medicine, Faculty of Health and Medical Sciences University of Copenhagen Copenhagen Denmark; ^5^ Lung Transplantation Unit, Department of Cardiology Copenhagen University Hospital – Rigshospitalet Copenhagen Denmark; ^6^ Neurovascular Research Laboratory, Faculty of Life Sciences and Education University of South Wales Pontypridd UK

In understanding the pathophysiology of kidney disease, as well as the mechanisms and effects of novel treatments targeting the kidney, the assessment of glomerular filtration rate (GFR) has served as an invaluable index of kidney function for nearly a century. The indirect clinical evaluation of GFR through the measurement of serum creatinine remains one of the most frequently requested blood tests in hospitals worldwide to this day. Its origins can be traced back to the Danish zoophysiologist Poul Brandt Rehberg (1895–1989), who worked as an assistant to August Krogh (1874–1949) in the 1920s.

Krogh himself had refrained from studying the kidney, describing it as a ‘devilish organ’ with reference to its inevitable complexity from a physiological perspective (Friis & Wang, 2017). However, as two opposing theories regarding kidney function – the filtration–absorption theory and the tubular secretion theory – remained unresolved at the time (Hoenig & Zeidel, 2014), he encouraged Rehberg to pursue this area of study. When Rehberg began his work, the tubular secretion theory was more widely accepted amongst contemporary scientists, as the sheer volume of blood that would need to be filtered and subsequently reabsorbed by the kidney appeared implausible, but it had recently been challenged by the seminal work *The Secretion of the Urine* (Cushny, 1917), by the Scottish physiologist and pharmacologist Arthur Cushny (1866–1926). To address this further, Rehberg turned his attention to the renal handling of creatinine, a substance already known as a naturally occurring by‐product of muscle metabolism that was excreted in urine, but at the time believed to exist only in trace amounts in the blood. Seeking to determine the relationship between creatinine excretion and glomerular filtration in the kidney, Rehberg devised an experimental approach that required repeated measurements in the same research subject. Despite his background as a zoophysiologist, Rehberg opted against using animal models for his research, stating that ‘the experiments made it necessary to obtain repeated series of observations on a single subject; it was almost impossible to use animals’ (Rehberg, 1926).

Following the Scandinavian tradition of self‐experimentation, particularly prominent in Krogh's Zoophysiological Laboratory as previously described in this journal (Berg, 2024, 2025), Rehberg conducted a series of experiments on himself – an early example of the now increasingly popular *n*‐of‐1 trial design! To achieve measurable plasma concentrations of creatinine, Rehberg ingested an oral dose of 5 g of creatinine and subsequently measured its levels hourly in both plasma and urine. Using these data, he derived a formula to estimate GFR based on creatinine clearance, demonstrating that his kidneys filtered approximately 180 L of fluid per day (Rehberg, 1926) – a finding that was somewhat unexpected, although Cushny had also provided GFR estimates (∼62 L per day) that were surprisingly high at the time, based on renal urea excretion (Cushny, 1917). While Rehberg's findings unequivocally supported the filtration–absorption theory, he was unable to entirely exclude the possibility that creatinine, in addition to glomerular filtration, might also undergo tubular absorption or secretion. And so the hunt for the ideal GFR tracer was on!

It was around this time that Homer W. Smith (1895–1962), who had recently been appointed Professor of Physiology and Director of the Physiological Laboratories at New York University School of Medicine, joined the quest. Smith's fascination with renal physiology could be traced back to his youth, when he was known to collect the urine of camels during circus visits to investigate how these animals could excrete such highly concentrated urine (Navar, 2004). He would ultimately become the uncontested patriarch of renal physiology, establishing the clearance technique as a powerful, non‐invasive method for obtaining mechanistic insights into kidney function. Smith had observed that aglomerular saltwater fish, such as the anglerfish (*Lophius*) and toadfish (*Opsanus*), were unable to excrete various carbohydrates (Shannon & Smith, 1935; Smith, 1937). He reasoned that these substances were not subjected to tubular absorption or secretion. Building on this observation, Smith hypothesised that inulin – a physiologically inert polysaccharide found in the roots of plants such as dahlias and Jerusalem artichokes – would undergo glomerular filtration alone. He subsequently employed its clearance from the blood to derive quantitative estimates of GFR in humans (Shannon & Smith, 1935; Smith et al., 1938).

Although Smith himself noted that the filter–reabsorption strategy ‘seemed extravagant and physiologically complicated’ (Smith, 1959), his work on the renal clearance of inulin and many other substances completely dismantled the tubular secretion theory. Through his integrative physiological perspective, he emphasised the kidney's critical role in maintaining body fluid homeostasis and regulating the composition of the *milieu intérieur* (Smith, 1953). Inulin clearance would ultimately become the undisputed reference method for measuring GFR. However, it was not without challenges. The protocol for measuring inulin clearance was both intricate and demanding, requiring an intravenous loading dose followed by a maintenance infusion to achieve stable plasma concentrations over a 24‐h period. This process necessitated precise timing for the collection of repeated blood samples and the catheterisation of the urinary bladder to collect urine. Such requirements rendered the protocol impractical in clinical settings.

In an effort to simplify the inulin clearance protocol, scientists from the Royal Infirmary in Edinburgh, as published in this journal – then entitled the *Quarterly Journal of Experimental Physiology and Cognate Medical Sciences* – reached a surprising conclusion that brought Smith's entire concept under serious scrutiny. By determining the renal clearance of inulin from the slope of the falling plasma curve of inulin concentration up to 150 min following a single injection in healthy males, they obtained results similar to those derived from the steady‐state inulin infusion method (Robson et al., 1949). However, in both cases, the measurements seemed to be biased by insufficient equilibration of inulin between the plasma and the extravascular compartment. Indeed, when inulin clearance was measured over several successive periods, it was observed that the values for clearance steadily declined as the plasma concentration of inulin fell, which pointed towards the possibility that inulin was being reabsorbed by the renal tubules to a significant extent (Ferguson et al., 1950). These findings suggested that the precondition for inulin clearance serving as a valid measure of GFR had been violated – one can only imagine the shock amongst contemporary renal physiologists! However, other scientists soon came to the rescue with counter‐evidence. They demonstrated that inulin clearance was, in fact, consistent across varying plasma concentrations even when using only a single injection, and emphasised the timing of measurements and related corrections as a substantial source of error, likely accounting for the observed discrepancies (Kennedy et al., 1953).

Inulin clearance, even when based on the single injection technique, still remained too laborious for widespread clinical application, as inulin is an inconvenient material to use, being poorly soluble in water and notoriously tedious to measure in the laboratory. While endogenous creatinine clearance techniques were developed based on Rehberg's work (Steinitz & Türkand, 1940), these proved too imprecise for many clinical situations (Dodge et al., 1967; Doolan et al., 1962; Kim et al., 1969). However, with the emergence of radioisotopes for diagnostic assessments and the broader development of the field of nuclear medicine, a method for measuring GFR based on the plasma clearance of ^51^Cr‐ethylenediaminetetraacetic acid (EDTA), as measured by the radioactive decay of ^51^Cr, was introduced. This method used the tracer kinetic principles developed by both Rehberg and Smith (Brøchner‐Mortensen, 1972; Brøchner‐Mortensen et al., 1969; Lavender et al., 1969; Stamp et al., 1970), and was later modified, such that a GFR measurement required a single plasma sample only (Mårtensson et al., 1998). This compound is not metabolised and is excreted exclusively through glomerular filtration in the kidneys, and although its clearance is slightly less than that of inulin due to a small degree of plasma protein binding and interactions with the negatively charged glomerular basement membrane, these differences were considered to fall within an acceptable margin of error for clinical purposes. This technique became the reference method for clinical GFR assessment, and it was by far the most widely used radioisotope‐based method for measuring GFR in the world until GE Healthcare ceased the production of ^51^Cr‐EDTA in 2020.

However, while both valid and reliable, ^51^Cr‐EDTA clearance and similar radionuclide‐based techniques, such as ^99m^Tc‐diethylenetriaminepentaacetic acid (DTPA) clearance, are limited to situations where highly accurate and precise GFR estimates are required. This limitation arises from the extensive set‐up necessary to obtain a single GFR value, including tracer injection and access to nuclear medicine facilities. Furthermore, such methods are unsuitable for routine screening or repeated assessments to evaluate dynamic changes within a short time frame. As a result, GFR assessment is much more commonly based on a single serum creatinine measurement. Several empirical equations exist to estimate GFR from such measurements, with the most widely used probably being the Chronic Kidney Disease Epidemiology Collaboration (CKD‐EPI) equation (Levey et al., 2009), which provides an estimated GFR (eGFR) based on serum creatinine, sex, age and previously also race. The inclusion of race in the equation was based on assumptions about race‐dependent differences in average muscle mass and creatinine generation. However, given that the concept of race is a sociopolitical construct rather than a biological entity as also discussed recently in this journal (Berg & Bailey, 2024; Cooper & Stanojevic, 2024; Lujan & DiCarlo, 2024; Yudell & Hammonds, 2024), a task force convened by the National Kidney Foundation and the American Society of Nephrology recommended removing race correction from GFR equations. This practice was deemed not only scientifically unfounded but also a contributor to health disparities between different ethnic groups, rather than a means of preventing them.

The CKD‐EPI equation suffers from several inherent limitations. Like other creatinine‐based equations, it is inextricably linked to the biological process of muscular creatinine generation and its relationship with renal clearance, the latter of which involves incremental tubular secretion as the GFR declines. Consequently, eGFR underestimates true GFR in highly muscular individuals and overestimates it in individuals with low muscle mass or when true GFR is low. Furthermore, the CKD‐EPI equation does not account for muscle mass or body size per se. This is particularly relevant given that CKD‐EPI indexes eGFR to a standardised body surface area (BSA) of 1.73 m^2^ for reporting purposes. The 1.73 m^2^ standard BSA was initially adopted as a matter of convenience based on estimated BSA measurements of young American men and women applying for life insurance in the 1920s (Heaf, 2007), but it should be noted that the CKD‐EPI equation does not directly consider any body proportions at all but merely estimates BSA based on age and sex. Alternative biomarkers of renal function, such as cystatin C, with corresponding regression equations to determine eGFR, have been developed. These biomarkers are less susceptible to the aforementioned confounders and are increasingly used worldwide (Spencer et al., 2023). Nevertheless, they are by no means routinely employed to the same extent as creatinine measurements and CKD‐EPI.

The lack of any direct estimates of body proportions constitutes a major caveat of the CKD‐EPI equation. Thus, when comparing eGFR with ^51^Cr‐EDTA clearance‐based GFR, the influence of muscle mass on eGFR turns out to be a major confounder. This was demonstrated in a study of 137 renal transplant patients, in which a clinically relevant absolute percentage error of more than 10% between the two methods was observed in over 70% of individuals (Nankivell et al., 2020). In a cohort of lung transplant patients followed at our own institution – initially studied primarily to investigate the extent and prognostic impact of clinically silent pulmonary thromboembolic disease (Kristensen et al., 2020; Mohammad et al., 2025) – we similarly observed that eGFR derived from CKD‐EPI often deviates critically and clinically significantly from ^51^Cr‐EDTA clearance‐based GFR. Given that this patient group is at high risk of renal failure, with post‐transplant immunosuppressive treatment being potentially nephrotoxic (Jing et al., 2021), GFR was routinely determined using ^51^Cr‐EDTA clearance until 2020 to ensure precise dosing. Over a period of 9 years, a total of 455 ^51^Cr‐EDTA clearance‐based GFR measurements were obtained at different time points in 247 patients. For 106 of these measurements, serum creatinine and concomitant eGFR calculated using the CKD‐EPI equation were obtained within the same 24‐h period. The findings, presented in Figure 1, indicate that when compared to ^51^Cr‐EDTA clearance, eGFR exhibited a clinically relevant 10% absolute percentage error in 79% of cases.

**FIGURE 1:**
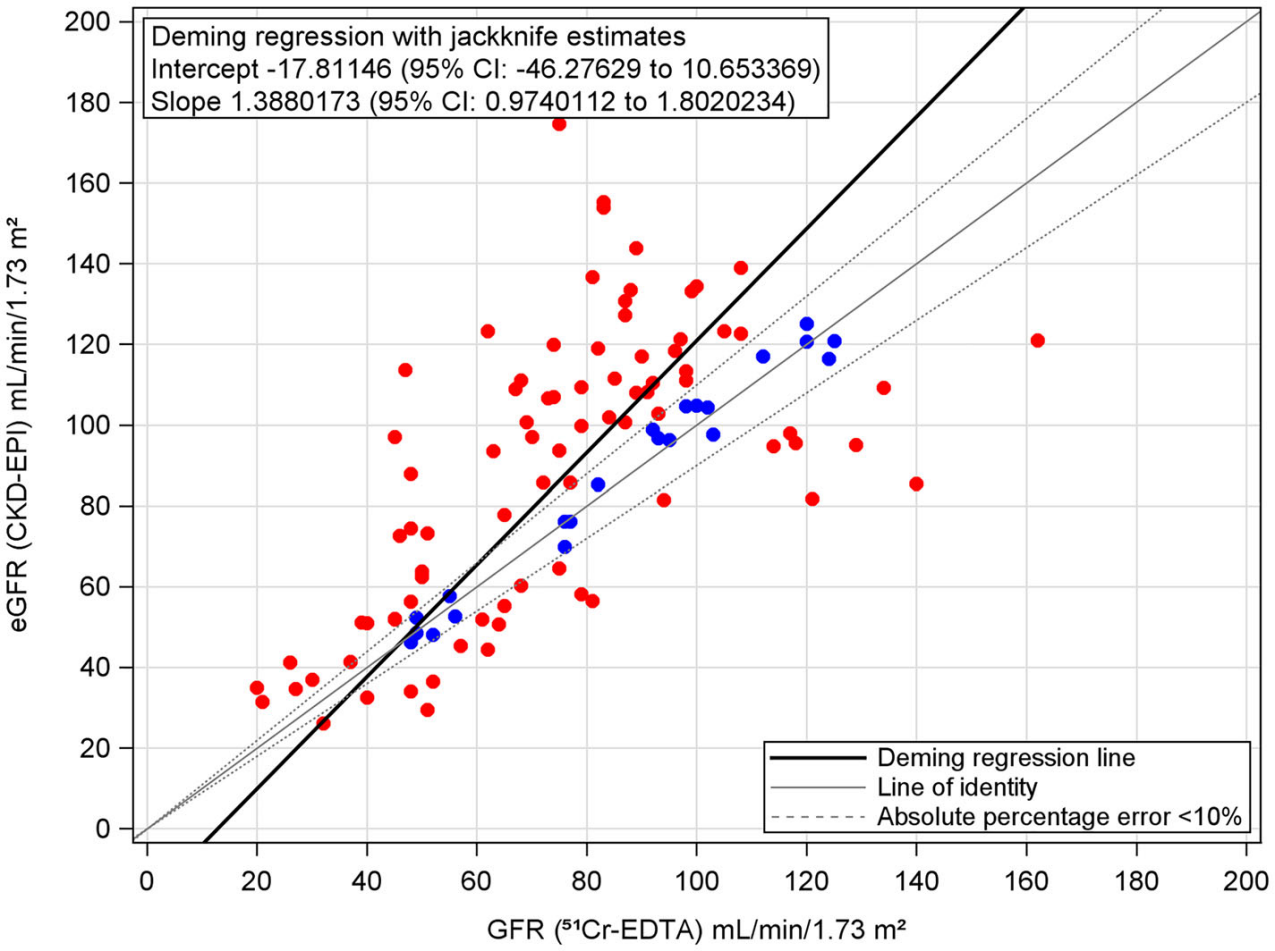
^51^Cr‐EDTA clearance‐based GFR versus eGFR based on the CKD‐EPI equation versus in lung transplanted patients (measurements: 106; individuals: 86); red ≥10% difference; blue: <10% difference. The agreement was calculated by Deming regression with standard error of estimates computed by the jackknife method (Linnet, 1993). CKD‐EPI, Chronic Kidney Disease Epidemiology Collaboration; eGFR, estimated GFR; GFR, glomerular filtration rate.

So, what can be learned from this Homerian odyssey into the *milieu intérieur*, which has taken us from Rehberg's classical work to the present‐day creatinine‐based regression equations? Perhaps it illustrates the difficulty of capturing the devilish and extravagant complexity of renal function in a single universal equation. However, one clear takeaway is that body proportions and composition matter, and it may be worthwhile to prioritise this consideration in the development of future creatinine‐based equations for estimating GFR.

## AUTHOR CONTRIBUTIONS

Anders Møller Greve: Conception, data collection, data analysis, revisions. Anna Agnes Lytze: First draft, revisions. Hrefna Sæunn Einarsdóttir: Data collection, revisions. Michael Perch: Revisions. Ronan M. G. Berg: Conception, first draft, supervision, revisions. All authors have read and approved the final version of this manuscript and agree to be accountable for all aspects of the work in ensuring that questions related to the accuracy or integrity of any part of the work are appropriately investigated and resolved. All persons designated as authors qualify for authorship, and all those who qualify for authorship are listed.

## CONFLICT OF INTEREST

None of the authors have any conflict of interest to declare.

## FUNDING INFORMATION

The Centre for Physical Activity Research (CFAS) is supported by TrygFonden (grants ID 101390, ID 20045, ID 125132, and ID 177225). The funders had no role in study design, data collection and analysis, decision to publish, or preparation of the manuscript.
